# *IDH* Mutation Analysis in Glioma Patients by CADMA Compared with SNaPshot Assay and two Immunohistochemical Methods

**DOI:** 10.1007/s12253-018-0413-9

**Published:** 2018-03-19

**Authors:** Irena Urbanovska, Magdalena Houdova Megova, Zachary Dwight, Ondrej Kalita, Magdalena Uvirova, Jarmila Simova, Lucie Tuckova, Petr Buzrla, Tomas Palecek, Marian Hajduch, Jana Dvorackova, Jiri Drabek

**Affiliations:** 1 CGB Laboratory Inc., Ostrava, Czech Republic; 2 Department of Biomedical Sciences, Faculty of Medicine, University of Ostrava, Ostrava, Czech Republic; 3 Institute of Molecular and Translational Medicine, Faculty of Medicine and Dentistry, Palacky University and University Hospital in Olomouc, Hnevotinska 5, 779 00 Olomouc, Czech Republic; 4 Department of Pathology, University of Utah, Salt Lake City, UT USA; 5 Department of Neurosurgery, University Hospital Olomouc, Olomouc, Czech Republic; 6 Department of Clinical and Molecular Pathology, Faculty of Medicine and Dentistry, Palacky University and University Hospital in Olomouc, Olomouc, Czech Republic; 7 Institute of Pathology, Faculty of Medicine and University Hospital, University of Ostrava, Syllabova 19, 703 00 Ostrava – Zábřeh, Czech Republic; 8 Neurosurgery Clinic, University Hospital Ostrava, Ostrava, Czech Republic

**Keywords:** *IDH1*, *IDH2*, CADMA, Glioma, Mutation testing

## Abstract

Mutations in *IDH1/2* genes are a marker of good prognosis for glioma patients, associated with low grade gliomas and secondary glioblastomas. Immunohistochemistry and Sanger sequencing are current standards for *IDH1/2* genotyping while many other methods exist. The aim of this study was to validate Competitive amplification of differentially melting amplicons (CADMA) PCR for *IDH* genotyping by comparison with SNaPshot assay and two immunohistochemical methods. In our study, 87 glioma patients (46 from Olomouc and 41 from Ostrava) were analyzed. *IDH1/2* mutations in native bioptical samples were analyzed at DNA level by CADMA and SNaPshot while IDH1 mutations in FFPE samples were analyzed at protein level by two IHC methods. CADMA PCR sensitivity for *IDH1* was 96.4% and specificity 100% for 86 concluded samples. SNaPshot assay sensitivity was 92.9% and specificity of 100% for 85 concluded samples. IHC in the laboratory no. 2 reached sensitivity 85.7% and specificity 100% for 86 concluded samples. IHC in the laboratory no. 4 reached sensitivity of 96.4% and specificity of 79.7% in 74 concluded samples. Only one *IDH2* mutation was found by SNaPshot while CADMA yielded false negative result. In conclusion, CADMA is a valid method for *IDH1* p.(R132H) testing with higher sensitivity than SNaPshot assay. Also, molecular genetic methods of *IDH1* testing from native samples were more robust than IHC from FFPE.

## Introduction

*IDH1/2* genes code for isocitrate dehydrogenases, catalysing the oxidative decarboxylation of isocitrate to α-ketoglutarate, resulting in the production of NADPH. In 2008, Parsons et al. found somatic mutations of isocitrate dehydrogenase 1 gene (*IDH1)* in glioblastomas [1]. *IDH1/2* somatic mutations are associated with lower normalized mean diffusion kurtosis [2], increased ZEB1 expression in lower-grade gliomas [3], preoperative seizures in gliomas [4], CpG methylator phenotype (CIMP), global hypermethylation, younger age, secondary glioblastoma, and increased overall survival [5, 6]. Mutations are considered to be an early event in gliomagenesis. Mutant IDH1/2 enzyme gains a new function – conversion of α-ketoglutarate (the normal product of wild type IDH enzymes) to 2 - hydroxyglutarate. This process consumes NADPH, disrupts cellular redox balance, induces mitochondrial instability [7], reprograms metabolism [8], affects DNA methylation [9, 10], and may be followed by IDH change from tumour driver to passenger [11].

The most frequent detection methods of IDH mutations are immunohistochemistry [12] and Sanger DNA sequencing [13] while wide spectrum of other DNA-based methods exists [14–21]. By now, Sanger sequencing has low limit of detection and next generation sequencing is prohibitively expensive. Thus, search for a reliable and robust gold standard assay for the *IDH* mutation is underway [22, 23].

In our study, we compared CADMA PCR method with SNaPshot assay and two immunohistochemical methods. Laboratories used their standard approach to *IDH1* testing: new application of Competitive amplification of differentially melting amplicons (CADMA) method (laboratory no.1), immunohistochemistry (laboratories no. 2 and no. 4), and SNaPshot assay (laboratory no. 3). Moreover, laboratories no. 1 and 4 performed also *IDH2* testing.

## Materials and Methods

Four laboratories from two different oncology centres of the Czech Republic (Olomouc and Ostrava) were included in an interlaboratory comparison of *IDH1* (NM_005896.3:c.395G > A, NM_005896.3(IDH1_i001):p.(Arg132His)) and *IDH2* (NG_023302.1:c.515G > A, p.(Arg172Lys)) mutation testing – laboratory 1: Institute of Molecular and Translational Medicine, Olomouc, laboratory 2: Department of Clinical and Molecular Pathology, Olomouc, laboratory 3: CGB laboratory, Inc., Ostrava, and laboratory 4: Institute of Pathology, Ostrava.

This retrospective case report was approved by an Ethical Board of Palacky University in Olomouc, NT 13581.

For interlaboratory comparison, 87 patient’s samples from patients who underwent surgery for glial tumour in Olomouc and/or Ostrava were used. No clinical informations were provided to laboratories before testing. Histologically, **87** glial tumours consisted of **2** pilocytic astrocytomas grade I, **9** diffuse astrocytomas grade II, **1** oligodendroglima grade II, **2** oligoastrocytomas grade II, **9** anaplastic astrocytomas grade III, **2** anaplastic oligodendrogliomas grade III, **3** anaplastic oligoastrocytomas grade III and **59** glioblastomas grade IV.

All patients in the study underwent brain biopsy or tumour resection between years 2007 and 2014 in Olomouc (*n* = 46, run A) and Ostrava (*n* = 41, run B). Subsequently, the material was processed at the laboratory no. 2 or laboratory no. 4 and was examined independently by two histopathologists to make the diagnosis according to the WHO classification 2007 [24]. During the paper preparation in 2016, the new guidelines for central nervous system tumours classification were established [25]. According to new classification, 87 patient’s samples consisted from 2 pilocytic astrocytoma, 9 diffuse astrocytomas, IDH-mutant, 1 oligodendroglioma, IDH-mutant and 1p/19q-codeleted, 2 oligoastrocytoma, IDH-mutant and 1p/19q codeleted, 8 anaplastic astrocytoma, IDH-mutant, 1 anaplastic astrocytoma, IDH-wildtype, 1 anaplastic oligoastrocytoma, IDH-mutant, 3 anaplastic oligodendroglioma, IDH-mutant and 1p/19q-codeleted, 5 glioblastoma, IDH-mutant, 55 glioblastoma, IDH-wildtype. Such reclassification does not affect analytical parameters of mutation testing methods.

**Fig. 1:**
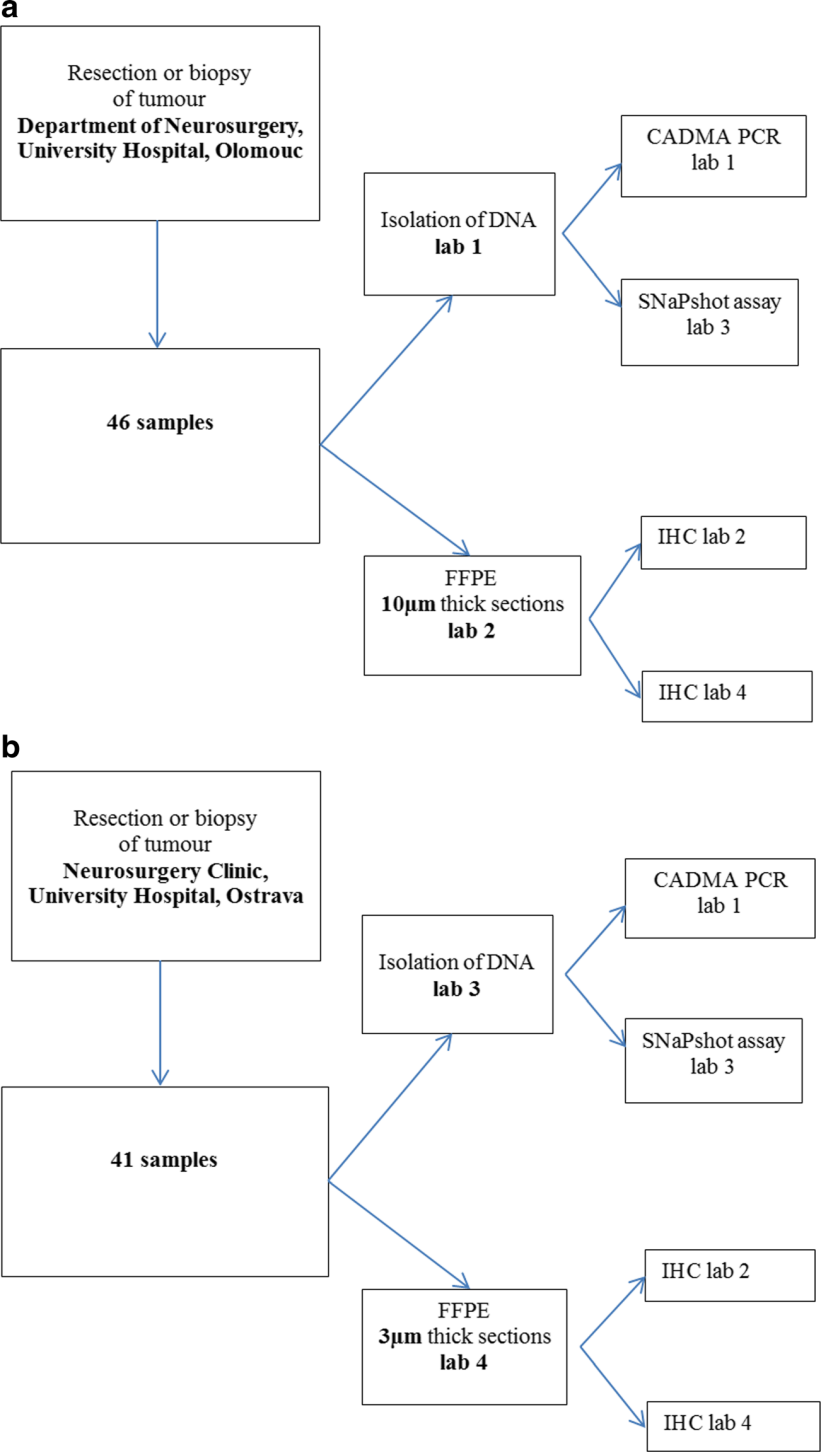
**a** Study design for 46 samples from Olomouc.Solid tumour tissue samples were obtained from patients who underwent surgery (resection or stereotactic biopsy) of brain for glial tumour (grade I-IV) in Faculty Hospital in Olomouc between years 2007-2013. The part of glial tissue sample in transporting medium (RPMI 1640 medium with L-glutamine, Penicilin/Streptomycin (100 U/ml), 15 % fetal bovine serum, insulin (100 IU/ml), transferrin (2 mg/ml), and heparin (25 000 IU/ml)) underwent transport at room temperature into laboratory and then was divided into smaller pieces that were frozen without any medium at -80°C for genomic DNA extraction and CADMA PCR. Another part of glioma tissue sample was fixed in 10% buffered formalin, than dehydrated in graded ethanol series, cleared by xylene, wax infiltrated, and paraffin embedded immediately after surgery (FFPE). 10 μm thick sections from FFPE samples were used for IHC in laboratory 4 and another 10 μm thick sections were used for IHC in laboratory 2. **b** Study design for 41 samples from Ostrava.Samples were obtained by resection or biopsy of tumour in Neurosurgery Clinic of University Hospital in Ostrava between years 2007-2014. Samples were divided in two parts; one part was inserted into a saline solution and sent to laboratory 3 for DNA isolation. SNaPshot assay was performed there and then an aliquot of DNA was sent to laboratory 1 for analysis by CADMA PCR. Second part of the bioptical sample was fixed in 10% buffered formalin as above. 3 μm thick sections from FFPE samples were used for IHC in laboratory 4 and another 3 μm thick sections were used for IHC in laboratory 2

Processing of samples in 4 laboratories is shown in diagrams in Fig. 1.

### Laboratory 1: CADMA Method

Genomic DNA purification was performed using Cobas DNA Sample Preparation Kit (Roche Diagnostics Corporation, Basel, Switzerland) according to the manufacturer’s instructions. Concentrations of DNAs were measured spectrophotometrically using NanoDrop ND 100 Spectrophotometer (NanoDrop Technologies, Wilmington, USA). Three CADMA PCR reactions (one for *IDH1* p.(R132H), one for p.(R132C) and one for *IDH*2 p.(R172K)) were performed per sample as previously been described [26].

### Laboratory 2: Immunohistochemistry

Ten-micrometer thick sections were pretreated 15 min at 120 °C to retrieve the antigen. The endogenous peroxidase activity was blocked using 6% H_2_O_2_. The sections were then incubated for 1 h with mouse monoclonal primary antibody against IDH-1 R132H (H09, 1:50, Dianova, Hamburg, Germany) and then with Dako EnVision+ Dual Link System-HRP secondary antibody (DAKO, Glostrup, Denmark) for 1 h at room temperature. The immunoreactivity was visualized by liquid DAB+ substrate-chromogen system (DAKO, Glostrup, Denmark). Finally, slides were washed under running water, dehydrated through graded ethanol and mounted. The nuclei were counterstained with hematoxylin.

### Laboratory 3: SNaPshot Assay

Genomic DNA purification of resection samples was performed using MagNA Pure Compact Nucleic Acid Isolation Kit (Roche Diagnostics) while bioptical samples were purified by NucleospinTissue (Macherey - Nagel, GmbH&Co.KG, Düren, Germany), according to manufacturers´ instructions. PCR for SNaPshot assay was performed using 1× PCR Master Mix (Thermo Scientific, Vilnius, Lithuania) and *IDH1* exon 4 primers as previously described [19]. The length (235 bp) and purity of PCR products were checked by electrophoresis on 3% agarose gel. 1 μl of PCR product was cleaned by Exo I – FastAP (Thermo Scientific, Vilnius, Lithuania) by incubating at 37 °C for 15 min followed by 80 °C for 15 min. Applied Biosystems® SNaPshot Multiplex Kit (Life Technologies, Woolston, Warington, UK) was used with 2 μl of cleaned PCR product and primers, specific for codon 132 of *IDH1* gene and codon 172 of *IDH2* gene as previously described [19], with following changes: SNaPshot PCR reactions were performed for 30 cycles of 96 °C for 10 s, 60 °C for 35 s, 10 μl of SNaPshot assay products were cleaned with FastAP incubating at 37 °C for 15 min folowed by 80 °C for 15 min, hold at 4 °C. 1 μl of cleaned SNaPshot assay products was mixed with 8.75 μl of Hi-Di formamide and 0.25 μl GS120LIZ Size ladder (Life Technologies, Foster City, CA, USA), denatured at 95 °C for 5 min and then separated on 3130 Genetic Analyzer with a 36 cm length capillary and POP-7™ polymer (Life Technologies), injection voltage 15 kV, injection time 8 s, run voltage 1.2 kV, oven temperature 60 °C and run time 450 s. The raw data from capillary electrophoresis were analysed on GeneMapper 3.7 Software (Life Technologies, Foster City, CA, USA).

### Laboratory 4: Immunohistochemistry

For immunohistochemical detection of IDH1 R132H protein, a double immunoperoxidase reaction was used. The 3 μm thick sections were pretreated 30 min at 100 °C to retrieve the antigen. IDH1 R132H Mouse anti Human unconjugated antibody clone H09 (Dianova GmbH, Hamburg, Germany) was diluted 1:20, applied to the pretreated paraffin sections and incubated for 30 min. Then, the secondary antibody EnVision (DAKO, Glostrup, Denmark) was used (1 h at room temperature) and the reaction was visualized with diaminobenzidine substrate (DAKO, Glostrup, Denmark).

**Fig. 2:**
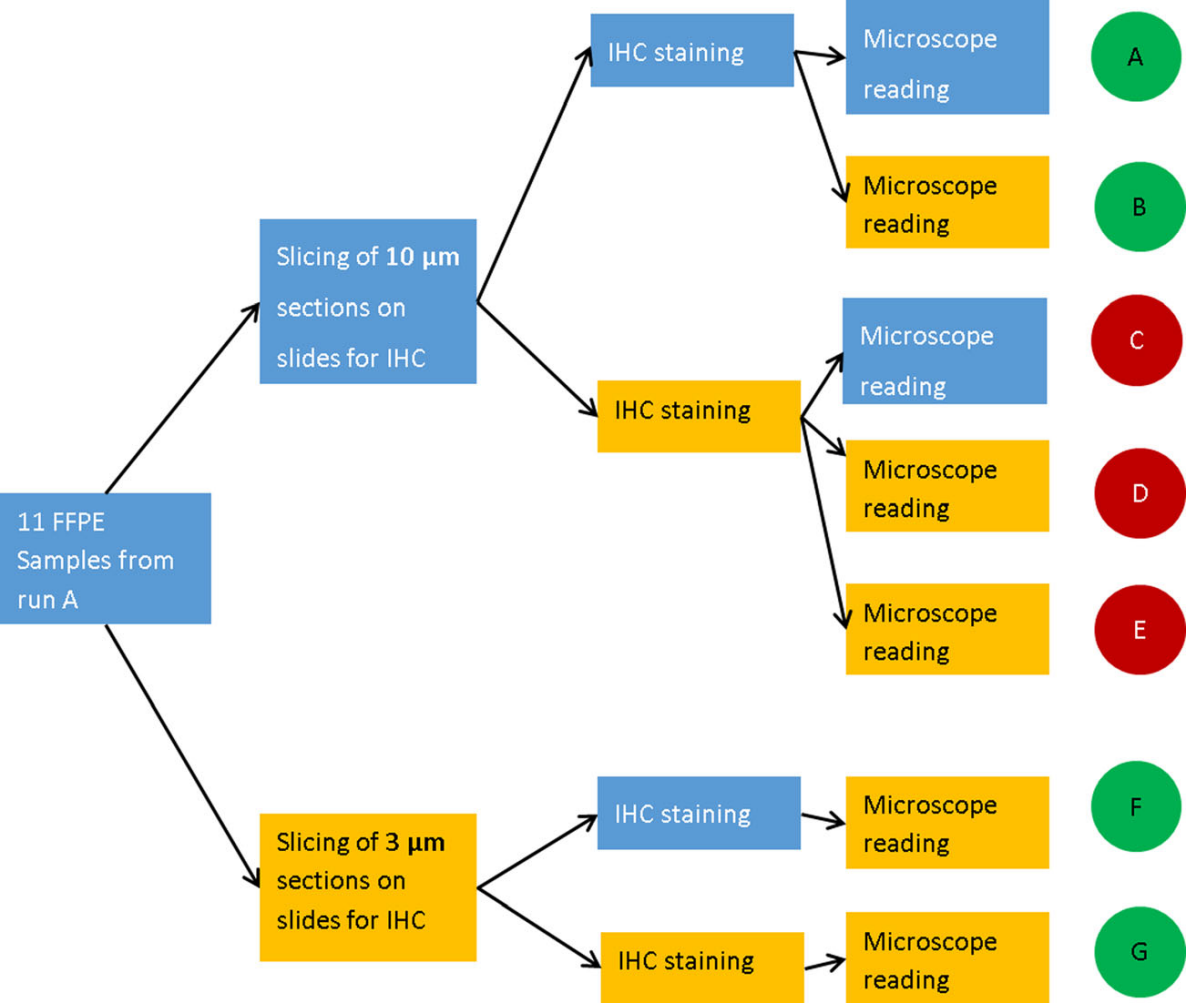
Study design for 11 discrepant samples that were analysed by IHC in detail (in pipelines A to G). Blue colour – procedure performed in laboratory no. 2, yellow colour – procedure performed in laboratory no. 4, red colour – false positive result, green colour – correct result

Discrepant samples that were available for further testing (11 samples from Olomouc) underwent further analysis showed on Fig. 2.

IHC slides were blindly rescored by independent neuropathologist. Then, FFPE blocks from laboratory no. 2 were sent to laboratory no. 4 and there were performed sectioning and IHC testing according to their methodology.

## Results

87 samples were tested for the presence of mutations in *IDH1* genes using four methods. Consensus *IDH1* results were reached by all four methods in 70 samples. 6 samples failed to determine the presence / absence of the mutation p.(R132C) by CADMA method. 23 samples were positive for mutation p.(R132H) and positivity was confirmed in all laboratories. 47 samples were negative for the presence of mutations p.(R132H) by all methods. Results of 17 samples were discrepant (Table 1).

**Table 1:** Discrepant results among four methods

	Source of material	Lab no. 1	Lab no. 2	Lab no. 3	Lab no. 4	Consensus
1	Lab no. 4	R132C	wt	R132C	wt	R132C
2	Lab no. 2	wt	wt	wt	R132H	wt
3	Lab no. 2	wt	wt	wt	R132H	wt
4	Lab no. 2	wt	wt	wt	R132H	wt
5	Lab no. 2	wt	wt	wt	R132H	wt
6	Lab no. 2	wt	wt	wt	R132H	wt
7	Lab no. 2	wt	wt	wt	R132H	wt
8	Lab no. 2	wt	wt	wt	R132H	wt
9	Lab no. 2	wt	wt	wt	R132H	wt
10	Lab no. 2	wt	wt	wt	R132H	wt
11	Lab no. 2	wt	wt	wt	R132H	wt
12	Lab no. 2	wt	wt	wt	R132H	wt
13	Lab no. 4	wt	wt	wt	R132H	wt
14	Lab no. 2	R132H	wt	R132H	R132H	R132H
15	Lab no. 4	R132H	wt	R132H	R132H	R132H
16	Lab no. 4	R132H	wt	wt	R132H	R132H
17	Lab no. 2	wt	R132H	wt	R132H	R132H

Among 17 discrepant samples, 1 sample was positive for the mutation p.(R132C) by molecular genetic methods; immunohistochemical methods could not determine this mutation using IDH1 p.(R132H) antibody.

12 samples at the laboratories no. 1, 2, and 3 were negative for the presence of mutations in the *IDH1* gene, laboratory no. 4 determined positivity. 11 samples came from Olomouc and retesting of discrepant samples was done. After retesting the consensus results were reached and all 11 samples were negative for presence of p.(R132H) mutation.

2 samples were positive for p.(R132H) mutation in three laboratories (laboratory no. 1, 3, and 4). In Laboratory no. 2 the positivity was not confirmed. 1 sample was positive for mutation p.(R132H) by CADMA method, this result was confirmed by immunohistochemistry in laboratory no. 4. Mutation was not detected in laboratory no. 2 and 3.

1 sample showed immunohistochemical positivity for p.(R132H) mutation, positivity was not confirmed by molecular genetic methods.

Laboratory no. 1 with CADMA PCR correctly concluded 86/87 samples (98.9%). In 81/87 samples, it was able to reach unambiguous PCR results for p.(R132H) and p.(R132C) mutation. 26 samples were positive for p.(R132H) mutation, 1 sample was positive for p.(R132C) mutation. 59 samples were negative and 1 sample was false negative for p.(R132H) mutation. In four p.(R132H) positive samples and two p.(R132H) negative samples, the test for p.(R132C) mutation failed. No *IDH2* R172K mutation was found (0/87).

For concluded samples, sensitivity was 96.4% and specificity 100%.

Laboratory no. 2 with IHC reached correct conclusion in 83/87 samples (95.4%). 24 samples were positive for p.(R132H) mutation, 59 samples were negative. 4 samples were false negative for presence of p.(R132H) mutation. For concluded samples, sensitivity was 85.7% and specificity 100%.

Laboratory no. 3 with SNaPshot assay correctly concluded 85/87 samples (97.7%). 25 samples were positive for p.(R132H) mutation, 1 sample was positive for p.(R132C) mutation. 59 samples were negative for presence of p.(R132H)/p.(R132C) mutation. 2 samples were false negative. For concluded samples, sensitivity was 92.9% and specificity of 100%. One *IDH2* R172M mutation was found (1/87).

Laboratory no. 4 with IHC reached correct conclusion for 74/87 samples (85%), with sensitivity of 96.4% and specificity of 79.7%. 39 samples were positive for p.(R132H) mutation, 12 samples of them were false positive. 47 were negative and 1 sample was false negative, because the antibody was not aimed to p.(R132C) epitope.

Consensus *IDH2* negative results were reached by two molecular genetics methods in 86 samples while 1 sample was discrepant between CADMA and SNaPshot. Sequencing analysis performed as previously described [16] of this sample confirmed mutation p.(R172M), revealed by SNaPshot.

Comparing molecular genetic methods for *IDH1* mutation analysis, identical results were achieved in 86 samples, 26 of them were positive, 60 samples were negative. One sample was false negative in laboratory no. 3 - SNaPshot assay did not detect positivity for p.(R132H) positive sample. Both immunohistochemical methods reliably determined the *IDH1* R132H positivity of the sample. One sample was false negative by both methods, positivity for p. (R132H) mutation was detected by both immunohistochemical methods.

Accordance between immunohistochemistry methods was achieved in 71 samples (24 were positive and 47 were negative); 3 samples were false negative in lab no. 2 and 1 sample was p.(R132C) false negative in both laboratories. Twelve samples were false positive in the laboratory no. 4. Such low specificity did not match the long term performance parameters of laboratory no. 4, as judged by results of external quality control. Therefore, the source of this discrepancy was analysed further. Eleven blocks originating from laboratory no. 2 (samples 2 to 12 in Table 1) were re-sliced, slides were re-stained, and re-read. The procedural combination of 10 μm slicing in laboratory no. 2 and IHC staining in laboratory no. 4 (reading C, D, and E) was found to be the source of discrepancy.

In sample 1, p.(R132C) mutation was detected by both molecular genetic methods while two IHC methods failed to find the mutation.

## Discussion

In our study, we compared CADMA PCR for testing of *IDH1/2* mutations with other molecular genetic method (SNaPshot assay) and 2 immmunohistochemical methods.

Pairwise comparison of molecular genetics methods for *IDH1* typing revealed one false negativity in SNaPshot assay. This can be explained by the difference in method sensitivities: while SNaPshot assay can detect only 5% of mutant DNA in the wild type background [19], CADMA PCR detects 2.5% of mutated alleles in a wild type background [26]. On the contrary, SNaPshot assay can detect larger spectrum of mutation in codon 132 of *IDH1* gene and is more robust with regards to quality of input DNA. The comparatively high failure rate of CADMA (6 samples) may be explained in 4 cases by decrease of effectiveness of p.(R132C) primers in p.(R132H) mutated template.

It remains to be seen if better sensitivity or better response rate and wider spectrum of detected variants bring better clinical value.

Pairwise comparison of IHC methods (laboratory 2 vs laboratory 4) showed higher response rate in laboratory 4 (100% vs 98.8%); however, this parameter did not translate into robustness of the assay. Laboratory 4 reached specificity of 79.7% while IHC in laboratory 2 (and molecular genetics methods) was 100% specific. We identified the root cause of the false positivity in laboratory 4 to be different thickness of FFPE sections from laboratory 2 (10 μm instead of 3 μm). Thicker sections were not compatible with antibody dilution (1:50 vs 1:20), antigen retrieval, processing, and interpretation of immunostaining in laboratory 4. This incompatibility probably caused differences in the relative impact of cytoplasmic staining, nuclear staining, and focal positivity on interpretation [12, 27, 28].

Our discrepancy in IHC methods is in contrast with finding of van den Bent et al. [23] who reported consistent results across IHC laboratories despite different terms of analysis. However, Preusser et al. admitted that in some cases, focal, weak, nonspecific background staining or regional heterogeneity of mIDH1-R132H protein expression is present and in these cases the confirmatory genetic testing may be necessary [23, 29].

We restrain from final conclusion about false negative result of CADMA for *IDH2* mutation testing till more *IDH2* R172K mutant samples are tested.

Pairwise comparison of molecular genetic methods vs IHC methods revealed false negativity of IHC methods in sample 1 of Table 1. This is not surprising as antibody was not aimed to p.(R132C) epitope. On the contrary, sample 17 in Table 1 revealed false negativity of molecular genetic methods. When DNA of this patient was isolated from FFPE sample, presence of mutation was confirmed by both molecular genetics methods and Sanger sequencing (performed as previously described [16]). Thus, the cause of discrepancy may have been the tissue heterogeneity within the native tumour sample when the p.(R132H) mutation was not present or its presence dropped below the detection limit of molecular genetic methods in the sampled part. The cause of the remaining discrepancies (samples 14, 15, and 16) is hard to be judged. It may be speculated that laboratory 2 failed to detect mutation in samples 14 and 15 due to inefficient staining of 10 μm slice.

Several authors found IHC testing of mIDH1 p.(R132H) protein more specific and sensitive than DNA sequencing [12, 23, 30] because sequencing detection limit is about 20% of mutant DNA on the wildtype background locus [19, 30] . Our molecular genetic methods gave more consistent results than IHC and are more sensitive than Sanger sequencing while their price is comparable with IHC. In the light of next generation sequencing developments, we propose to stop the Sanger sequencing to be considered gold standard of genotyping. Both molecular genetic methods used the same DNA from the sample and it seems that results of both methods are independent of type of DNA extraction. Nevertheless, DNA was isolated from native tumour samples. If the DNA was isolated from FFPE samples, then number of successfully analysed samples would drop and inconclusive results would increase due the presence of the PCR inhibitors and the quality DNA which is degraded by histopathological processing [31, 32].

Also, validated IHC methods are quick and do not require specific equipment [30, 33]. IHC is able to detect p.(R1321H) mutation in a single infiltrating cell [12]. However, detection of other mutations on 132nd residue of *IDH1* gene requires specific antibodies [34] that were not used in this study.

On the contrary, molecular genetic methods, especially SNaPshot assay, are able to detect more mutations in codon 132, but require expensive chemistry and equipment, and results are dependent on DNA quality and on immunohistochemical assessment of the proportion of mutated cells.

In our study, the comparison of CADMA PCR with two IHC methods and one molecular genetics method (SNaPshot) was done. CADMA PCR was validated and found to be performing at least as analytically as other methods. Contrary to previous findings, molecular genetic methods showed higher concordance and higher sensitivity but were more affected by a low quality of sample. IHC methods were affected by laboratory-based differences in pre-analytical phase. If any of four tested methods was performed from preferred input material and standard procedure was followed from pre-analytical phase, then their results are comparable.
